# Early-life exposure to severe famine is associated with higher methylation level in the *IGF2* gene and higher total cholesterol in late adulthood: the Genomic Research of the Chinese Famine (GRECF) study

**DOI:** 10.1186/s13148-019-0676-3

**Published:** 2019-06-10

**Authors:** Luqi Shen, Changwei Li, Zhenghe Wang, Ruiyuan Zhang, Ye Shen, Toni Miles, Jingkai Wei, Zhiyong Zou

**Affiliations:** 1 Department of Epidemiology and Biostatistics, College of Public Health, University of Georgia, Health Sciences Campus, 101 Buck Road, Athens, GA 30602 USA; 2 Institute of Child and Adolescent Health, School of Public Health, Peking University, 38 Xue Yuan Road, Haidian District, Beijing, 100191 China; 3 Department of Epidemiology, School of Global Public Health, University of North Carolina at Chapel Hill, 135 Dauer Drive, Chapel Hill, NC 27599 USA

**Keywords:** DNA methylation, Chinese Famine, HDL-C, LDL-C, Triglycerides, Total cholesterol, Mediation analyses

## Abstract

**Objective:**

To evaluate the association of early-life exposure to the Chinese Great Famine (1959–1961) with DNA methylation in *IGF2* and its subsequent influence on blood lipid levels in late adulthood among participants of the Genomic Research of the Chinese Famine (GRECF) study.

**Methods:**

The GRECF study recruited 790 participants born between 1956 and 1964 from 2 neighbor provinces, Anhui and Jiangxi, in China through a multistage, clustered, random sampling. The current study included a random sample of 188 GRECF participants. *IGF2* differential methylation region (DMR) is an intragenic DMR located upstream of the imprinted promoters of *IGF2* exon 3. DNA methylation were quantified at 8 cytosine-phosphate-guanine dinucleotides (CpG) sites at the *IGF2* DMR (chr11p15.5) using the Sequenom EpiTYPER method and the MassARRAY system. Multivariate linear regressions were used to evaluate pairwise associations among famine severity, DNA methylation in the *IGF2* gene, and lipid levels. We controlled for age and sex in the base model and additionally controlled for education, smoking, and drinking status in the fully adjusted model. Mediation analysis was applied to assess the mediation effect of DNA methylation at the *IGF2* gene on the association between early-life exposure to severe famine and adult lipid levels.

**Results:**

Exposure to severe famine was associated with elevated methylation at CpG1 (chr11: 2126041, build 36) of the *IGF2* DMR (*β* = 0.07; *P* = 0.0008) and total cholesterol (*β* = 0.72; *P* = 1.09 × 10^−7^). After adjustment for age and sex, each unit increase in methylation of the CpG1 site was associated with 1.09-unit increase in total cholesterol (*P* = 0.03). After further adjustment for all covariates, these associations were still significant (*P*_famine-CpG1_ = 0.002, *P*_famine-total cholesterol_ = 1.28 × 10^−6^, and *P*_CpG1-total cholesterol_ = 0.05).

**Conclusion:**

Increased methylation level in the *IGF2* gene was associated with early-life exposure to severe famine, and this change was also positively associated with total cholesterol in late adulthood.

**Electronic supplementary material:**

The online version of this article (10.1186/s13148-019-0676-3) contains supplementary material, which is available to authorized users.

## Introduction

Malnutrition during childhood has been associated with a series of health problems indicated by high mortality, morbidity, and mental disability [1–6]. Because of ethical concern, the common practice in epidemiology, such as a malnutrition exposure experiment, has not been and should not be applied to human beings. Alternatively, some natural disasters, such as famine, were widely studied as an ideal (quasi-) natural experiment to investigate the long-term effect of childhood malnutrition. In studies of the Dutch famine in 1944–1945, individuals exposed to famine up to 6 months during gestation was associated with elevated total cholesterol (TC), triglycerides (TG), low-density lipoprotein cholesterol (LDL-C), LDL-C to high-density lipoprotein cholesterol (HDL-C) ratio, and apolipoprotein B, and lower levels of HDL-C and apolipoprotein A [7–9]. Similar studies of the Chinese Great Famine (1959–1961) indicated that early-life exposure to severe famine had excessive risk of dyslipidemia [10]. Furthermore, the Dutch famine study identified that associations of prenatal undernutrition with elevated total cholesterol concentrations and triglycerides only existed among women, but not men [7]. The phenomenon was also found in studies of the Chinese Great Famine [10].

An explanation for these findings is that famine may cause life-long changes in DNA demethylation [11–13] and subsequently increase blood lipid levels in late life [10]. The Dutch famine studies have reported that participants exposed to famine up to 6 months during gestation had different methylation level in late adulthood (> 60 years) at the *IGF2*, *INSIGF*, *IL10*, *LEP*, *ABCA1*, *GNASAS*, and *MEG3* gene loci, compared to their sex-matched siblings without famine exposure [12, 13]. In a recent publication, Tobi and colleagues further demonstrated that DNA methylation at 6 genes mediated associations of prenatal famine exposure with triglycerides, an important component of blood lipids [14]. However, the findings are lacking replication in an independent sample.

Compared to the Dutch famine, the Chinese Great Famine lasted much longer and had greater severity. The Chinese Great Famine occurred between 1959 and 1961. According to our previous research, about 11.6% of the middle-aged and older Chinese adults had family member(s) starve to death during that period [15]. In addition, post-famine food supplies were not fully recovered until 20 years later in the 1980s [16]. Therefore, DNA methylation changes in the above mentioned genes should be more prominent and cause larger effect in lipid profiles among individuals exposed to the Chinese Great Famine. Study of the Chinese Great Famine provides a unique opportunity to replicate findings in the Dutch famine studies and, more importantly, may reveal novel associations between methylation in those genes and lipid profiles.

Therefore, we conducted the first study to explore the associations of early-life exposure to the Chinese Great Famine with blood lipids and to assess the mediation effect of methylation in *IGF2* gene, the best-characterized epigenetic loci, on the famine-lipids association in a Chinese population.

## Materials and methods

### Data source and study participants

The Genomic Research of the Chinese Famine (GRECF) study was designed to evaluate the impact of the Chinese Great Famine on human genome and the subsequent consequences on metabolic disorders. The GRECF study was conducted between July 2015 and January 2016 among residents of Anhui and Jiangxi provinces, China. The 2 provinces were chosen as study sites because of their large difference in famine severities and similarity in geographic location, ethnic composition, lifestyle, and dietary habits. Historically, famine was most intense in Anhui province and moderate in Jiangxi province [17, 18]. According to our previous research, 35.5% of the middle-aged and older adults in Anhui province reported having immediate family members starve to death during the Chinese Great Famine in 2011 [15]. Jiangxi province neighbors Anhui province, but only 6.8% of the middle-aged and older residents reported death of family members due to the famine [15]. In addition, the economic development, predominantly agricultural during 1960s, in the 2 provinces was also similar. Therefore, it is reasonable to assume that the 2 provinces would have experienced a similar temporal trend regarding population health, if there was no famine.

Participants of the GRECF study were recruited through a multi-stage, clustered, random sampling. Specifically, in the first stage, 1 well-developed region and 1 developing region were randomly selected in each province. In the second stage, 3 counties and 3 villages in each region were randomly selected. Finally, 30–35 subjects born in the 3 years immediately before (1956–1958), during (1959–1961), or immediately after (1962–1964) the Chinese Great Famine were randomly selected from each county or village based on birthdate obtained from the National Resident Registration System. Overall, a total of 790 participants consented to participate and completed the anthropometric assessments and survey questionnaires.

Due to limited budget, DNA methylation was profiled among a random sample of 188 participants from GRECF participants who experienced famine as infants or fetus and from those who were born after the famine. The sampling process was stratified by regions, birth cohorts, and gender. In each region-birth cohort-gender stratum, one third of participants were randomly selected to have their DNA methylation at the *IGF2* gene assayed. The current analyses were performed among those 188 participants.

### Famine exposure and covariates in the GRECF study

Food shortages gradually occurred in late 1958 [19]. For the purpose of this study, we select the start date of the Chinese Great Famine as January 1, 1959, and the end date as December 31, 1961. Therefore, early-life famine exposure was categorized into 2 levels: severe and moderate. Specifically, individuals born in Anhui province were categorized as having severe famine exposure, and participants born in Jiangxi province were considered as having moderate famine exposure.

Age was obtained from the National Resident Registration System. Other demographic and health behavioral information, including gender, education levels, smoking, and drinking, were based on self-report. Education levels were categorized into “no more than elementary school,” “middle school,” “high school,” and “some college or above.” Smoking status was categorized as “currently smoking” or “currently not smoking.” Drinking status was classified as “a current drinker” or “not a current drinker.”

### DNA methylation in the IGF2 gene and blood lipid measurement

Blood samples were collected between August 2015 and May 2016, about 5 decades after the famine. A 4-mL tube of fasting blood sample was collected for each participant. A unique barcode was generated for each participant and attached to the blood tubes. After collection, the fresh venous blood samples were transported at 4 °C temperature to Peking University Health Science Center, where all samples were separated into plasma and buffy coat and stored at − 80 °C for use. Genomic DNA was extracted from peripheral white blood cells using the salting-out method [20]. The EZ 96-methylation kit (Zymo Research) was used to treat DNA with bisulfite. The Sequenom MassARRAY system (Sequenom, San Diego, CA) was used to quantify DNA methylation levels at 8 cytosine-phosphate-guanine dinucleotides (CpG) sites of the *IGF2* gene using the manufacturers’ protocol on a 384-well plate. Primers of *IGF2* gene was designed using the Epidesigner online application (http://epidesigner.com/start3.html). Details of the primers are presented in Additional file 1: Table S3. CpG sites are numbered according to the position of its cytosine in the amplicon counting from p-ter onwards.

DNA methylation of the *IGF2* gene was profiled using the EpiTYPER, a mass spectrometry-based bisulfite sequencing method that enables region-specific DNA methylation analysis in a quantitative and high-throughput fashion [21]. The EpiTYPER has high precision and high inter-lab reproducibility and can detect down to 5% changes in methylation levels. In the measurement of *IGF2* DNA methylation, we strictly followed an EpiTYPER protocol designed specifically for the *IGF2* gene, which had stringent quality assurance and quality control measures [22]. Specifically, all reaction agents were from the same batch of products, and standard quality control samples and process blank were used throughout the methylation quantification process. More importantly, all samples were assayed in triplicate. If the triplicate methylation measurements had an extreme value equal to or greater than 3 standard deviation (SD), all data for the sample involved were discarded. The mean success rate for the 8 successfully assayed CpG sites was 87.0%. Among the 8 CpG sites, 6 CpG sites were measured individually, and 2 were measured simultaneously because they could not be resolved due to their close proximity. DNA methylation at a CpG site is quantified as a beta value, which is the ratio of methylated signal intensity to the sum of methylated signal intensity, unmethylated signal intensity, and an offset constant.

Blood lipids were assayed from fasting blood samples at the same time as the blood DNA methylation was collected. TC, LDL-C, and HLD-C were assayed using enzymatic colorimetric tests. TG was calculated using the Friedman formula and was logarithmically transformed for analyses.

### Statistical analysis

Means and standard deviations for continuous and frequencies and percentages for categorical characteristics were calculated for the overall participants and by severities of famine. Continuous variables were checked for normality and log-transformed if necessary. Bivariate association analyses were conducted between famine severity and all covariates using chi-square tests for categorical variables and one-way analysis of variance (ANOVA) for continuous variables.

The associations of famine severity with lipids and DNA methylation at the *IGF2* gene were assessed by multivariate linear regressions adjusting for age and sex. After Bonferroni correction, significant CpG sites in the *IGF2* gene were further tested for associations with lipid levels in multivariate linear regression models adjusting for age and sex. To test the robustness of the associations, we additionally controlled for education levels, smoking, and drinking status in the fully adjusted models. Finally, we quantified the mediation effect of DNA methylation at the significant CpG site of the *IGF2* gene as the proportion of total famine-lipids relationship mediated by DNA methylation at the significant CpG site of the *IGF2* gene. The mediation analysis used the classic 2-regression approach described by Baron and Kenny [23]. Sobel’s test of significance was performed to determine the extent to which CpG1 site contributed to the total effect on TC [24]. Models were adjusted for age, gender, education level, smoking status, and drinking status."

To reduce potential misclassification of famine severities, sensitivity analyses were performed excluding individuals born within 3 years after the famine. All analyses were performed using the SAS software (version 9.4; SAS Institute Inc., Cary, North Carolina). Two-sided *P* values were provided, and *P* < 0.05 was considered significant. A *P* value of 0.007 was used to determine significant CpG site (correcting for 7 independent CpG sites).

## Results

Characteristics of study participants are presented in Table 1. Participants were on average 54.2 years old. Less than half (48.4%) of the participants were males. Only 30.9% of the participants had high school or more education. Prevalence of current smoking was 36.7%, and 40.4% were current drinkers. Those in severe famine group were on average 0.7 years older than those in moderate famine group. There were no significant differences in gender, education levels, smoking, or drinking status between the 2 groups.

**Table 1:** Characteristics of the GRECF study participants by levels of famine severity

Covariates	Overall (*n* = 188)	Famine experience level		*P*
		Moderate (*n* = 83)	Severe (*n* = 105)	
Male, *N* (%)	91 (48.4)	43 (51.8)	48 (45.7)	0.41
Age, years, mean (SD)	54.2 (2.3)	53.8 (2.4)	54.5 (2.2)	0.0001
Education levels, *N* (%)				
No more than elementary school	58 (30.9)	30 (36.1)	28 (26.7)	0.21
Middle school	72 (38.3)	28 (33.7)	44 (41.9)	
High school	49 (26.1)	19 (22.9)	30 (28.6)	
Some college and above	9 (4.8)	6 (7.2)	3 (2.9)	
Current smoker, *N* (%)	66 (36.7)	23 (30.7)	43 (41.0)	0.16
Current drinker, *N* (%)	76 (40.4)	29 (34.9)	47 (44.8)	0.17
LDL, mmol/L, mean (SD)	2.9 (0.8)	2.6 (0.6)	3.2 (0.9)	7.91 × 10^−7^
HDL, mmol/L, mean (SD)	1.3 (0.3)	1.2 (0.3)	1.3 (0.4)	0.007
logTG, mean (SD)	0.4 (0.5)	0.4 (0.5)	0.5 (0.5)	0.08
TC, mmol/L, mean (SD)	4.9 (0.9)	4.4 (0.7)	5.2 (0.9)	1.64 × 10^−9^

*SD* standard deviation, *LDL-C* low-density lipoprotein cholesterol, *HDL-C* high-density lipoprotein cholesterol, *logTG* logarithmically transformed triglycerides, *TC* total cholesterol

Multiple adjusted associations between exposure to severe famine, DNA methylation, and lipid levels in adulthood were illustrated in Tables 2–4. After controlling for age and gender, individuals with severe famine exposure in early life had significantly higher TC (*β* = 0.72, *P* = 1.09 × 10^−7^), LDL-C (*β* = 0.55, *P* = 1.03 × 10^−5^), and HDL-C (*β* = 0.10, *P* = 0.04) in late adulthood (Table 2), compared to those with moderate famine exposure in early life. The associations were stronger among females (Table 2); however, gender differences were not statistically significant. The 2 groups had similar levels of log transformed TG (*β* = 0.12, *P* = 0.12) (Table 2). Meanwhile, early-life severe famine exposure was significantly associated with higher DNA methylation level at CpG1 site in the *IGF2* gene. When stratified by gender, the DNA methylation level at CpG1 site in the *IFG2* gene was most pronounced among female offspring (*β* = 0.10, *P* = 0.004) (Table 3). After controlling for age and gender, exposure to severe famine in early life was associated with 7% (*P* = 0.0008) increase in DNA methylation level at the CpG1 site, compared to those who experienced moderate famine (Table 3). When restricting to participants born during or before the famine, the associations of famine severity with DNA methylation at the CpG1 site and TC were still significant (Additional file 1: Tables S1 and S2). Finally, DNA methylation at the CpG1 site was also positively associated with TC (Table 4). Per unit increase of DNA methylation in the CpG1 site, age- and sex-adjusted TC increased by 1.09 mmol/L (*P* = 0.03). No gender difference was observed in the association of this CpG site with TC (*P* for interaction = 0.70). Furthermore, mediation analysis demonstrated that the mediation path through the CpG1 explained 5% (*P* = 0.30) of the association between famine severity and TC in adulthood.

**Table 2:** Association of exposure to severe famine with lipids among GRECF study participants

		Age- and sex-adjusted model*		*P*_interaction_^†^	Fully adjusted model**		*P*_interaction_§
		Beta ( SE)	*P*		Beta( SE)	*P*	
HDL-C	Overall	0.10 (0.05)	0.04	0.17	0.11 (0.05)	0.04	0.30
	Male	0.03 (0.07)	0.63		0.04 (0.07)	0.56	
	Female	0.17 (0.07)	0.02		0.15 (0.08)	0.06	
LDL-C	Overall	0.55 (0.12)	1.03 × 10^−5^	0.55	0.52 (0.12)	3.71 × 10^−5^	0.90
	Male	0.47 (0.17)	6.26 × 10^−3^		0.48 (0.17)	5.79 × 10^−3^	
	Female	0.62 (0.17)	5.65 × 10^−4^		0.53 (0.18)	4.48 × 10^−3^	
logTG	Overall	0.12 (0.08)	0.12	0.41	0.10 (0.08)	0.22	0.52
	Male	0.20 (0.12)	0.11		0.16 (0.13)	0.23	
	Female	0.04 (0.10)	0.68		0.09 (0.11)	0.42	
Total cholesterol	Overall	0.72 (0.13)	1.09 × 10^−7^	0.98	0.67 (0.13)	1.28 × 10^−6^	0.74
	Male	0.73 (0.19)	2.48 × 10^−4^		0.68 (0.20)	1.01 × 10^−3^	
	Female	0.71 (0.18)	1.57 × 10^−4^		0.63 (0.19)	1.09 × 10^−3^	

*LDL-C* low-density lipoprotein cholesterol, *HDL-C* high-density lipoprotein cholesterol, *logTG* logarithmically transformed triglycerides, *SE* standard error

*Adjusted for age and sex among the overall sample; adjusted for age among male and female, respectively

^†^Assessed by adding an interaction term, gender × famine severity to the age- and sex-adjusted model among the overall participants

**Adjusted for age, sex, smoking, drinking, and education level among the overall sample

^§^Assessed by adding an interaction term, gender × famine severity to the fully adjusted model among the overall participants

**Table 3:** Association of exposure to severe famine with CpG1 site of the *IGF2* gene among GRECF study participants

		Age- and sex-adjusted model*		*P*_interaction_†	Fully adjusted model**		*P*_interaction_§
Methylation		Beta( SE)	*P*		Beta( SE)	*P*	
Overall	Overall	0.005 (0.01)	0.70	0.89	0.004 (0.01)	0.75	0.91
	Male	0.005 (0.02)	0.78		0.003 (0.02)	0.87	
	Female	0.006 (0.02)	0.76		0.003 (0.02)	0.88	
CpG1 site	Overall	0.07 (0.02)	*0.0008*	0.18	0.07 (0.02)	*0.002*	0.15
	Male	0.04 (0.02)	0.09		0.03 (0.03)	0.24	
	Female	0.10 (0.03)	*0.004*		0.10 (0.04)	*0.008*	
CpG2 site	Overall	− 0.01 (0.02)	0.82	0.56	− 0.01 (0.03)	0.78	0.73
	Male	0.005 (0.03)	0.88		− 0.002 (0.04)	0.95	
	Female	− 0.01(0.04)	0.72		− 0.008 (0.04)	0.84	
CpG3 site	Overall	0.02 (0.02)	0.42	0.08	0.02 (0.02)	0.41	0.07
	Male	0.05 (0.03)	0.04		0.06 (0.03)	0.03	
	Female	− 0.02 (0.03)	0.54		− 0.02 (0.03)	0.45	
CpG4 site	Overall	− 0.02 (0.02)	0.27	0.71	− 0.02 (0.02)	0.24	0.52
	Male	− 0.01 (0.02)	0.54		− 0.01 (0.02)	0.63	
	Female	− 0.03 (0.03)	0.36		− 0.04 (0.03)	0.22	
CpG5 site	Overall	0.01 (0.01)	0.24	0.64	0.01 (0.01)	0.29	0.83
	Male	0.008 (0.01)	0.33		0.005 (0.01)	0.53	
	Female	0.007 (0.01)	0.47		0.01 (0.01)	0.33	
CpG6 and CpG7 site	Overall	− 0.02 (0.01)	0.13	0.24	− 0.02 (0.01)	0.15	0.38
	Male	− 0.004 (0.02)	0.78		− 0.002 (0.02)	0.91	
	Female	− 0.04 (0.02)	0.11		− 0.04 (0.02)	0.12	
CpG8 site	Overall	− 0.02 (0.02)	0.44	0.56	− 0.02 (0.02)	0.27	0.75
	Male	− 0.005 (0.02)	0.83		− 0.019 (0.02)	0.41	
	Female	− 0.03 (0.03)	0.43		− 0.04 (0.03)	0.26	

*SE* standard error

*Adjusted for age and sex among the overall sample; adjusted for age among male and female, respectively

^†^Assessed by adding an interaction term, gender × famine severity to the age- and sex-adjusted model among the overall participants

**Adjusted for age, sex, smoking, drinking, and education level among the overall sample

^§^Assessed by adding an interaction term, gender × famine severity to the fully adjusted model among the overall participants

**Table 4:** Association between CpG1 site of the *IGF2* gene and lipids among GRECF study participants

		Age- and sex-adjusted model*		*P*_interaction_^†^	Fully adjusted model**		*P*_interaction_^§^
		Beta( SE)	*P*		Beta (SE)	*P*	
LDL-C	Overall	0.70 (0.44)	0.12	0.50	0.63 (0.44)	0.15	0.64
	Male	0.24 (0.79)	0.76		0.22 (0.76)	0.77	
	Female	0.89 (0.55)	0.11		0.71 (0.55)	0.20	
HDL-C	Overall	0.20 (0.18)	0.27	0.87	0.17 (0.18)	0.35	0.97
	Male	0.16 (0.31)	0.60		0.17 (0.33)	0.60	
	Female	0.22 (0.23)	0.34		0.11 (0.23)	0.64	
logTG	Overall	0.18 (0.28)	0.52	0.46	0.14 (0.29)	0.63	0.38
	Male	− 0.12 (0.56)	0.83		− 0.21 (0.56)	0.71	
	Female	0.31 (0.30)	0.31		0.39 (0.31)	0.22	
Total cholesterol	Overall	1.09 (0.49)	0.03	0.70	0.94 (0.48)	0.05	0.79
	Male	0.84 (0.91)	0.36		0.66 (0.89)	0.46	
	Female	1.20 (0.57)	0.04		1.00 (0.58)	0.09	

*LDL-C* low-density lipoprotein cholesterol, *HDL-C* high-density lipoprotein cholesterol, *logTG* logarithmically transformed triglycerides, *SE* standard error

*Adjusted for age and sex among the overall sample; when stratified by gender, age was adjusted among male and female, respectively

^†^Assessed by adding an interaction term, gender × CpG1 to the age- and sex-adjusted model among the overall participants

**Adjusted for age, sex, smoking, drinking, and education level among the overall sample; when stratified by gender, age, smoking, drinking, and education level were adjusted among male and female, respectively

^§^Assessed by adding an interaction term, gender × CpG1 to the fully adjusted model among the overall participants

## Discussion

To our knowledge, this is the first epigenetic study of early-life exposure to the Chinese Great Famine and blood lipids in later life. Our study supports a finding from studies of the Dutch famine that early life famine exposure is associated with methylation in the *IGF2* gene. Furthermore, we provide a novel evidence that methylation in the *IGF2* gene was positively associated with TC in late adulthood. The findings not only help to understand the function of *IGF2* but aid in delineating the mechanisms of cholesterol metabolism.

The identified associations of early-life famine exposure with blood lipids are in line with a previous population-based study that individuals who experienced severe famine in their early-life had higher LDL-C in later life than those who experienced moderate famine [10]. Our study provided further evidence that early-life severe famine exposure was also positively associated with TC.

The *IGF2* gene encodes insulin-like growth factor II and plays a key role in human development and growth. Animal studies revealed that *lgf2* knockout mice mimicked nutritional deficiency in utero and resulted in growth restriction and neonatal glycogen stores reduction [25]. The gene is maternally imprinted and paternally expressed [26–28], and methylation changes in the gene persist until at least middle age [29]. Demethylation of the DMR is linked to loss of imprinting [28, 30] and subsequently leads to overexpression of *IGF2* [31]. The multifaceted effects of *IGF2* and methylation plasticity of its DMR have led many, including our group, to study how diverse early-life environmental conditions influence methylation of this region [13, 32] and its subsequent relations to many metabolic disorders [30, 31, 33]. Prenatal environmental exposures have important role in the DNA methylation level of imprinted genes including the *IGF2*. Previous studies have demonstrated that prenatal exposure to maternal stress and anxiety was negatively associated with methylation level in the DMR of *IGF2* [34, 35]. On contrary, in Mexican-American newborn children, prenatal phthalate and estradiol exposures increased methylation of the *IGF2* DMR [36, 37]. Nutrition supply during pregnancy also influences DNA methylation in the IGF2 gene. Prenatal high-fat and high-sugar diet was associated with higher *IGF2* methylation [38]. Our study demonstrated that early-life exposure to the Chinese Great Famine up to 3 years was associated with hyper-methylation in the *IGF2* among human, which can inhibit the expression of the *IGF2* gene [39] and may cause growth restriction. Interestingly, the Dutch famine study showed that prenatal exposure to famine for 6 months was associated with lower methylation in the *IGF2* gene [13]. Meanwhile, methylation in the *IGF2* gene was not associated with birth weight in the Dutch famine studies [13]. Therefore, we hypothesize that transient exposure to famine may cause hypomethylation of the gene and trigger growth in utero, while long-term extreme famine exposure may cause hypermethylation in the gene and restrict growth in utero. The difference between our study and the Dutch famine studies may also be attributed to several other factors, such as differences in genetic backgrounds, dietary habits, post-famine food supply, and war experience. Besides distinct genetic backgrounds, people in the Netherlands have a diet relatively rich in animal proteins [40, 41], while the Chinese diet is mainly composed of carbohydrates [42]. During the Dutch famine, the distribution of energy intake in the percentage of calories from proteins, fat, and carbohydrates was 12%, 19%, and 69%, respectively [40, 12]. The proportion of energy intake in the percentage of calories from proteins, fat, and carbohydrates was 9.5–9.7%, 5.5–7.6%, and 82.9–84.8% in the Chinese Famine [42]. In addition, food supply in China was not fully recovered until 20 years later in 1980s, while food supply was immediately resolved after the Dutch famine. Finally, the Chinese Great Famine occurred in a no-war period; therefore, confounding effect of stress due to war experience in the Dutch famine survivors may also contribute to the difference.

The current study also identified that DNA methylation at the CpG1 site of the *IGF2* gene was positively associated with TC in late adulthood. To our knowledge, this is the first study showing association of DNA methylation of the *IGF2* with TC. Similar trends were observed for LDL-C and HDL-C. However, due to small sample size and limited power, we could not find significant associations between the CpG1 and the other 2 lipid components. In previous study, DNA methylation in the *IGF2* was positively associated with TG, but not TC among 85 children [43]. TG is more likely to be influence by daily diet, suggesting that variations of the TG is even larger, which can further reduce power to detect an association between the CpG1 and TG in this study. Although further studies are needed to validate the finding in an independent Chinese sample, our study together with the Dutch famine studies indicates that the *IGF2* gene is important in lipid metabolism in human. The finding is supported by a recent study among mice, which demonstrated that *Igf2* knockout mice displayed altered expression levels of genes involved in lipid and fatty acid metabolisms [25]. Early-life nutrition supply, development and growth, and lipids in adulthood warrant further investigation.

Finally, differential methylation at the CpG1 unit of *IGF2* contributed to a fairly small proportion (5%) of the association between famine severity and adult TC levels. Still, a large proportion of famine-TC association remains unexplained. Future genome-wide epigenetic studies are warranted to identify more methylation regions underlying such association.

Our study represents the first epigenetic research on the early-life exposure to the Chinese Great Famine and lipid levels in late adulthood. The current study has several advantages. First, the timing (occurred 59 years ago), length (lasted for 3 years), and scale (all of mainland China) of the Chinese Great Famine provided a rare opportunity to study the impact of long-term extreme nutrition deprivation during early life on lipid levels in late adulthood. Although a humanitarian disaster in China, famine mimicked a natural experiment of nutrition deprivation and provided an unparalleled opportunity to study changes in DNA methylation due to environmental influence (famine). Second, the Chinese Great Famine happened in a no-war period and a strictly governed society where migration was prohibited. Consequently, it is exempted from some potentially confounding factors, such as psychological stress associated with war and violence experience. Third, most previously studied famine happened in wealthy societies, in which residents suffered from temporary starvation and then recovered with abundant food supplies right after the famine. In contrast, even after the 1959–1961 famine, food supply was limited (although improved to extent degree) for couple decades in China until the market economy reform in 1980s. Therefore, epigenetic changes due to extreme malnutrition during early-life stage can be more likely maintained till later life. As we expected, the Chinese Great Famine was associated with greater changes (7%) in DNA methylation at the *IGF2* CpG1 site in this study compared with that (3.4%) in the Dutch famine study. Additionally, we were able to identify associations of early-life exposure to the Chinese Great Famine with LDL and TC not only among women but also man and overall participants, which were missed in the Dutch famine [7, 12]. Several limitations should be mentioned. First, DNA methylation data was measured from peripheral whole blood, which might be not the most relevant tissues to study for lipids, and the cellular heterogeneity of whole blood may contribute as a potential noise. However, previous DNA methylation data from multiple tissues from the same individual showed DNA methylation patterns of the CpG units in whole blood were similar to various fat deposits [44, 45]. Second, the statistically significant mediation observed in the current study did not clarify the mechanism, but it does provide possible pathways to explore in future. Third, blood lipids and DNA methylation of the *IGF2* gene were measured at the same time. The temporal sequence of methylation and lipid changes is unclear. However, DNA methylation of the *IGF2* gene is stable and largely driven by genetic factors rather than age-related environmental or stochastic factors [29, 46]. The *IGF2* per se is involved in lipid metabolism. Animal studies identified that disruption of the *Igf2* gene alters hepatic lipid homeostasis and gene expression in mice [25]. Negative feedback loop regulation of lipids on *IGF2* expression has not been reported. Therefore, it is likely that methylation changes in *IGF2* gene results in lipids change. Still, the probability that DNA methylation of the *IGF2* gene is partially due to high cholesterol cannot be fully ruled out. Future longitudinal analyses are warranted to delineate the temporal relationship. Forth, cell-type heterogeneity was not corrected in the current study. Compositions of white blood cells change with aging, and cell-type-specific methylation patterns may cause false positive findings. However, Talens and colleagues demonstrated that variations in *IGF2* DNA methylation were not associated with cell-types in whole blood samples [47]. Furthermore, methylation in the *IGF2* gene measured from whole blood samples is highly correlated with that in other tissues [47]. Therefore, we did not correct for cell-type heterogeneity in the current study. Previous study showed that a 10-year older age was generally associated with a 3.6% lower methylation [13]. In our study, participants who were exposed to severe famine was 0.7 years older and had higher DNA methylation level, indicating that, if cell-type heterogeneity contributes to *IGF2* methylation variation, we may slightly underestimate the methylation level in the exposed group. There should be a larger difference between the exposed group and the reference group if age was perfectly balanced. This also indicates that our finding is robust. Finally, compared to the huge body of Chinese adults who experienced the Chinese Great Famine, our sample size is very small; therefore, sample variation should be large, and this may limit the generalizability of our study findings. Future Chinese Great Famine studies with larger sample sizes are warranted.

## Conclusion

In the present study, we found that increased methylation level in the *IGF2* gene may be a consequence of early-life exposure to severe famine during the Chinese Great Famine and this change was also positively associated with TC in late adulthood.

## Additional file

Additional file 1: Supplementary tables. This file contains supplementary **Tables S1–S3.** (DOCX 18 kb)

## Abbreviations

ANOVA: One-way analysis of variance
CpG: Cytosine-phosphate-guanine dinucleotides
GRECF: Genomic Research of the Chinese Famine
HDL-C: High-density lipoprotein cholesterol
LDL-C: Low-density lipoprotein cholesterol
TC: Total cholesterol
TG: Triglycerides
