# Supplementary material for: Early-life exposure to severe famine is associated with higher methylation level in the IGF2 gene and higher total cholesterol in late adulthood: the Genomic Research of the Chinese Famine (GRECF) study
Source: Clin Epigenetics. 2019 Jun 10;11:88. doi: 10.1186/s13148-019-0676-3 (PMC6558811; doi:10.1186/s13148-019-0676-3)
Supplement: Supplementary file 1 — Supplementary tables. This file contains supplementary Tables S1–S3. (DOCX 18 kb) [file 13148_2019_676_MOESM1_ESM.docx]

**Supplementary Table S1. Association of Exposure to Severe Famine with Lipids Levels excluding GRECF Study Participants born before famine.**

|  |  | Age, sex adjusted model | |  | Fully adjusted model^[[1]](#footnote-1)^ | |
| --- | --- | --- | --- | --- | --- | --- |
|  |  | Beta( SE) | *P* |  | Beta( SE) | *P* |
| HDL-C |  | 0.11 (0.07) | 0.09 |  | 0.12 (0.07) | 0.07 |
| LDL-C |  | 0.68 (0.15) | 2.08×10^-5^ |  | 0.62 (0.15) | 8.31×10^-5^ |
| logTG |  | 0.18 (0.10) | 0.07 |  | 0.17 (0.11) | 0.12 |
| Total cholesterol |  | 0.86 (0.17) | 1.00×10^-6^ |  | 0.79 (0.17) | 7.55×10^-6^ |

LDL-C =Low-density lipoprotein cholesterol; HDL-C =High-density lipoprotein cholesterol; TG=Triglycerides; SE=standard error

**Supplementary Table S2. Association of Exposure to Severe Famine with CpG1 site of the IGF2 gene excluding Study Participants born before famine.**

|  |  | Age, sex adjusted model | |  | Fully adjusted model^[[2]](#footnote-2)^ | |
| --- | --- | --- | --- | --- | --- | --- |
|  |  | Beta( SE) | *P* |  | Beta( SE) | *P* |
| CpG1 site |  | 0.07 (0.03) | 0.02 |  | 0.07 (0.03) | 0.03 |

SE=standard error

**Supplementary Table S3. Details of the measured amplicons and the PCR primers.**

| **CpG sites in the current study** | **Genomic**  **location (b36)** | **CpG sites in Dutch Famine study** | **Function** | **Primer** |
| --- | --- | --- | --- | --- |
| *IGF2* | chr11: 2126035-2126372 |  | Differentially methylated region (DMR), an intragenic DMR located upstream of the imprinted promoters of *IGF2* exon 3 associated with IGF2 expression, imprinted | *Forward:* TGGATAGGAGATTGAGGAGAAA  *Reverse:* AAACCCCAACAAAAACCACT |
| CpG1 | chr11: 2126041 | CpG1 |  |  |
| CpG2 | chr11: 2126057 | CpG2 and CpG3 | PAX-6 binding site |  |
| CpG3 | chr11: 2126060 |  |  |  |
| CpG4 | chr11: 2126154 | Excluded for rs4930041 |  |  |
| CpG5 | chr11: 2126202 | CpG4 |  |  |
| CpG6 | chr11: 2126251 | CpG5 |  |  |
| CpG7 | chr11: 2126296 | Excluded for rs3741209 |  |  |
| CpG8 | chr11: 2126312 | Excluded for rs3741208 and rs17883577 |  |  |

1. Adjusted for age, sex, education levels, smoking, drinking among the overall sample. [↑](#footnote-ref-1)
2. Adjusted for age, sex, education levels, smoking, drinking among the overall sample. [↑](#footnote-ref-2)
